# Effect of distribution of educational material to mothers on duration and severity of diarrhoea and pneumonia, Midlands Province, Zimbabwe: a cluster randomized controlled trial

**DOI:** 10.1186/s13006-015-0037-6

**Published:** 2015-03-27

**Authors:** Meggie Gabida, Milton Chemhuru, Mufuta Tshimanga, Notion T Gombe, Lucia Takundwa, Donewell Bangure

**Affiliations:** Department of Community Medicine, University of Zimbabwe, Harare, Zimbabwe; Provincial Medical Directorate, Midlands Province, Ministry of Health and Child Care, Harare, Zimbabwe

**Keywords:** cIYCF, Exclusive breastfeeding, Promotion, Mother-based

## Abstract

**Background:**

Exclusive breastfeeding rates remain low in most countries in sub-Saharan Africa. We assessed the effects of a mother-based intervention on duration of diarrhoea and pneumonia in communities that were trained and those not trained in community infant and young child feeding (cIYCF) in Midlands Province, Zimbabwe.

**Methods:**

We evaluated communities with village health workers who received training in cIYCF and the distribution of educational materials (newsletter) to mothers in promotion of exclusive breastfeeding using a two-by-two factorial cluster randomized controlled trial. The trial arms included clusters trained in cIYCF only, clusters with mothers that received a newsletter only, clusters that received both interventions and clusters receiving no intervention. Consenting mother-infant pairs identified within 72 hours of delivery were followed up at 14 and 20 weeks where duration of diarrhoea and pneumonia as well as severity of diarrhoea was assessed. Clusters were facility catchment areas assigned by an independent statistician using randomization generated by a computer using Stata 10. All admitting facilities and facilities at borders were excluded as buffer zones and eight clusters were analysed. Nutritionists who collected data were not aware of the hypothesis being tested and analysis was by intention-to-treat.

**Results:**

A total of 357 mother-infant pairs were available for analysis in all the clusters. The interaction between cIYCF training and the newsletter was statistically significant at 14 weeks (p = 0.022). The mean duration of diarrhoea was 2.9 (SD = 0.9) days among infants of mothers who resided in communities trained and received a newsletter compared to 5.2 (SD = 1.1) days in communities that received neither. The protective efficacy of the cIYCF plus newsletter was 76% during the first 20 weeks of life. In the two way ANOVA, the newsletter was more effective on duration of pneumonia (p = 0.010) at 14 weeks and remained significantly effective at 20 weeks (p < 0.0001).

**Conclusions:**

A combined community and distribution of a newsletter to mothers on promotion of exclusive breastfeeding reduces duration of diarrhoea at 14 weeks. At 20 weeks, the newsletter worked better for both duration of diarrhoea and pneumonia compared to cIYCF training alone.

## Background

Exclusive breastfeeding has been estimated to reduce infant mortality rate by 13% during the first six months of the baby’s life [1,2]. Exclusive breastfeeding means that an infant receives only breast milk with no additional foods or liquids and not even water for the first six months of life; vitamin supplements, minerals and medicines are also not given unless medically indicated [3,4]. Breast milk provides all the energy and nutrients that the infant requires for the first six months of life. It continues to provide one third of the child’s nutritional needs during the second year of life [3,4]. United Nations Children’s Fund (UNICEF) reiterates that despite compelling evidence that exclusive breastfeeding (EBF) prevents diarrhoea and pneumonia, global rates remains stagnant in the developing world growing from 32% in 1995 to 39% in 2010 [2,3,5,6].

Exclusive breastfeeding (EBF) also provides health benefits for mothers [3]. Breastfeeding contributes to maternal health in the immediate postpartum period by helping the uterus to contract rapidly, thereby reducing blood loss. In the short term, breastfeeding delays a woman’s return to fertility and in the long term it reduces the risk of cancers of the breast and ovary [3,4].

Globally, it is estimated from 94 countries reported under World Health Organization (WHO) Nutrition Data Bank that out of the 65% of the world’s infant population, (<12 months) 35% are exclusively breastfed between zero and four months of age [5]. Therefore one in every three children is exclusively breastfed in the developing world. WHO and UNICEF thus set several global strategies aiming to improve breastfeeding. These include; the global strategy for infant and young child feeding (IYCF), the baby friendly hospital initiative (BFHI) and the international code of marketing of breast milk substitutes [6-8].

The global strategy on infant and young child feeding emphasizes on optimal infant and child feeding practices where infants should be exclusively breastfed for the first six months of life and continue to breastfeed from six months with timely, adequate complementary foods up to two years or beyond [1,6,7]. The Baby Friendly Hospital Initiative (BFHI) is the translational tool developed by WHO and UNICEF to promote breastfeeding in maternity wards worldwide [6,7]. It implements the ten steps to successful breastfeeding which are the corner stone to child survival. BFHI recommends early initiation of breastfeeding within the first hour of birth, where early initiation is putting the baby to the breast within one hour of delivery [7]. On the other hand, international code of marketing of breast milk substitutes in a country bans the advertisement of breast milk substitutes [8]. It protects mothers and caregivers from pressures of commercial foods advertisements idealizing breast milk substitutes thereby encouraging mothers to breastfeed their children.

Among the global initiatives is the commemoration of the world breastfeeding week (WBW) that takes place every first week of August [9]. Various activities are carried out in the world according to the theme for that year, educating communities while promoting, protecting and supporting breastfeeding.

In the recent years, the Human Immune Deficiency Virus (HIV) epidemic became a major challenge to EBF. However, there are large disparities between continents with the developed world almost eliminating mother-to-child transmission of HIV [10]. WHO’ s recent guidelines on infant feeding and HIV recommends that mothers infected or uninfected with HIV or with unknown status should practice EBF for the first six months, introducing appropriate complimentary foods thereafter and continuing to breastfeed up to 24 months or beyond [11]. Breastfeeding should stop after a nutritionally adequate diet without breast milk can be provided [7,10], otherwise the mother should continue breastfeeding up to 24 months.

In sub-Saharan Africa the enormous benefits of EBF include improved nutrition and reductions in infant morbidity, mortality and mother-to-child transmission of HIV (MTCT) [11]. EBF seems to have a protective effect on HIV-1 transmission compared to mixed feeding [10-12]. Both the HIV-1-positive women in resource-poor settings and the overall population might therefore benefit from this practice [5,10,11]. Women living with HIV can reduce vertical HIV transmission to their children through practicing EBF for the first six months of the baby’s life.

Major causes of under-five mortality in Zimbabwe are HIV/AIDS, pneumonia and diarrhoea [12,13]. The nation is significantly below target to meet millennium development goals (MDGs) 4 and 5 [12]. Countrywide, EBF rates have remained almost stagnant. According to the Zimbabwe Demographic Health Survey 2010–11, the rates have risen slightly from 22% in 1999 to 31% in 2010. The low EBF rates strongly correlate with stunting levels, under-five mortality and infant mortality. Against this background, Zimbabwe is not on track to achieve MDG 4 [12].

Midlands province has a total population of 1 602 733. Of these, 241 051 (15%) are children under five years [14]. And of the under five years, 48 563 (20.1%) are under one year who had a median duration of EBF of only 1.2 months according to the ZDHS 2010–11 report [12]. Most of the districts in the province have stunting levels above 30% [12-14]. The province hosted the national world breastfeeding week (WBW) commemoration in August 2010. Since then, the province embarked on extensive scale up of community infant and young child feeding trainings (cIYCF) in a bid to increase community participation in improving infant feeding practices, particularly EBF.

Gweru and Kwekwe are neighbouring districts that share boundaries. Children under five in the two districts constitutes about 13% and 14% respectively of the total population. The prevalence of diarrhoea in under-fives is 13.2% in the province [12] and the largest population in the two districts resides in rural communities. Most (98%) of the children, below the age of six months receive some breast milk but not exclusively [12]. Some reasons cited for failure to exclusively breastfeed are that mothers believe they have inadequate milk and that children cry excessively because they will be hungry [12,13,15,16].

Nationally it is of great concern that only 5.8% of Zimbabwean children were exclusively breastfed as of 2010. During the same year in Midlands Province, only about 6% of children were exclusively breastfed whereas Gweru, Kwekwe and Gokwe South districts recorded 0% exclusive breastfeeding rates [13]. The MDGs target for exclusive breastfeeding is at least 70%. Against this background, about 94% and 100% of children in Midlands province and Gweru district respectively; had they been exclusively breastfed could have been protected from infectious diseases such as pneumonia and diarrhoea (ranked number one and two causes of under-five morbidity and mortality) respectively [14,16,17].

This study assessed the effects of distribution of educational material to mothers (mother-based promotion of exclusive breastfeeding) on duration of diarrhoea and pneumonia. The findings of the study will complement the Ministry of Health and Child Care, Zimbabwe in their efforts to promote and increase exclusive breastfeeding rates.

## Methods

### Study setting

The setting was two districts in Midlands Province which lies in the southern region of Zimbabwe. All rural health facilities and urban clinics in the two districts (Gweru and Kwekwe) were included in the study. According to the ZDHS 2010–11 report; 71.1% of women in the province can read and only 4% cannot [12]. Rural health facilities and urban clinics provide primary care services to surrounding communities and each has a catchment population of approximately 10,000 people. The facilities are manned by at least three primary care nurses in rural or registered general nurses in urban settings, one environmental health technician and two other auxiliary staff. In each district ideally health facilities/clinics should be within eight kilometre (km) radius but majority are 20 to 60 km apart or more. Within each catchment area of a clinic there is support of village health workers trained by the Ministry of Health and Child Care that enhance capacity for provision of preventive and health promotion interventions at community level.

### Study design

**Figure 1:**
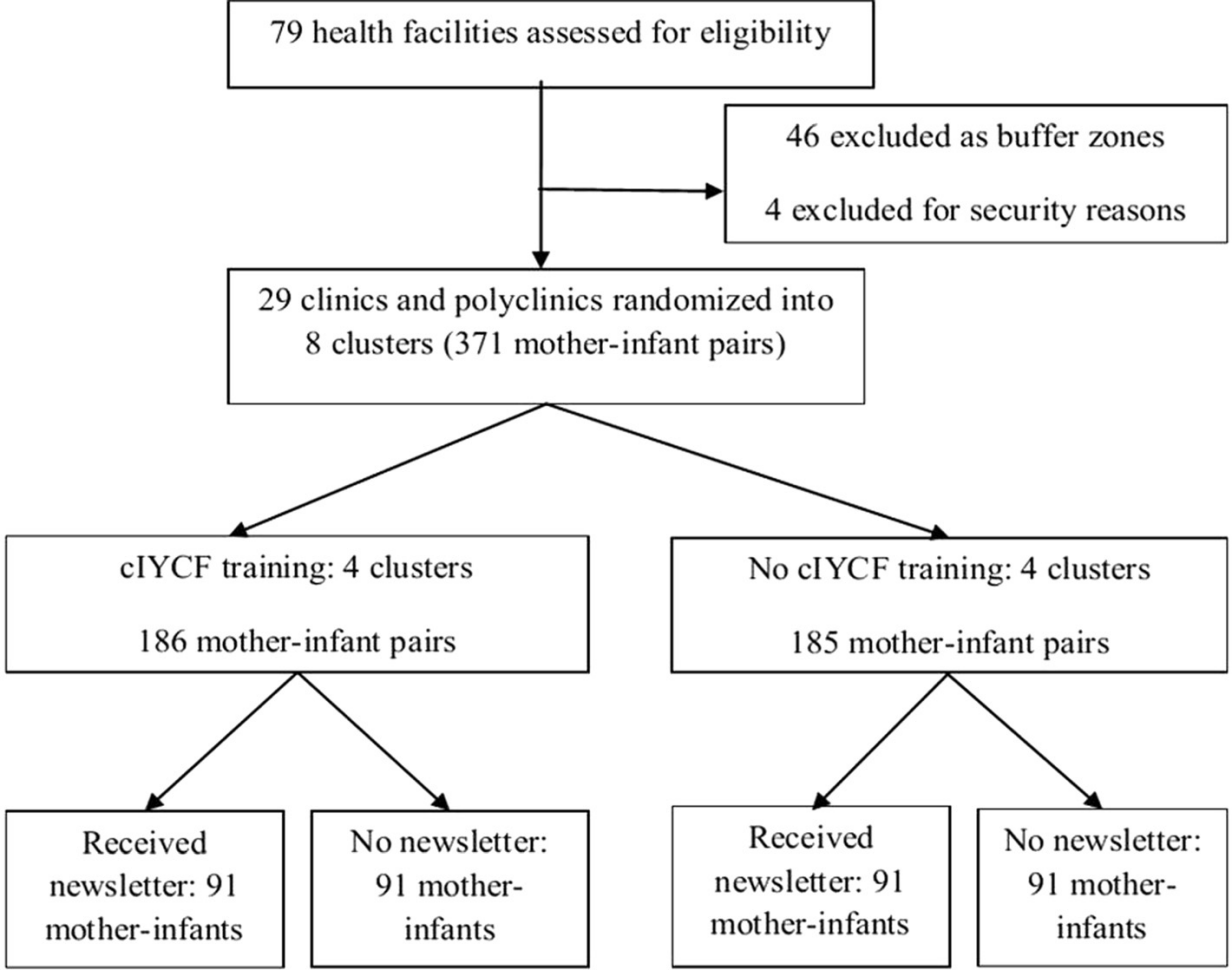
**Trial profile.**

A cluster randomized controlled trial was conducted where the clusters were neighbouring health facilities (clinics) serving an average catchment area of 10 000 people in the rural and urban communities of Gweru and Kwekwe districts. The cluster design was chosen because the unit of randomization was a group rather than an individual to minimize contamination. A 2 by 2 factorial design was used to further randomize clusters into one of four trial arms so as to assess if there was an interaction between the independent variables. The trial arms included clusters trained in cIYCF only, clusters with mother-infant pairs that received a newsletter only, clusters trained in cIYCF and mother-infant pairs received a newsletter (both interventions) and clusters with infant-mother pairs receiving no intervention (Figure 1).

### Study sample

Mothers registered or unregistered in antenatal care register who delivered at facilities or at home within the selected clusters and these mother-infant pairs were identified within 72 hours of delivery by the nurses and village health workers between December 2012 and February 2013.

### Target group and inclusion criteria

The target population was communities living 10 kilometres to nearest health facility. Clusters of clinics and polyclinics in Gweru and Kwekwe district were included. Only mothers who delivered within selected clusters and identified within 72 hours of delivery and informed written consent obtained were recruited into the study. Ability of the mother to read and residing within 10 km radius of the nearest health facility were also considered.

### Exclusion criteria

Admitting hospitals and clinics at the borders of clusters were excluded as buffer zones. Infants with mothers who chose not to breastfeed, and did not reside permanently in the selected clusters were not included in the study.

### Ethical approval

Permission to carry out the study was obtained from the Joint Parirenyatwa Hospital and College of Health Sciences Research Ethics Committee (JREC) and; the Medical Research Council of Zimbabwe (MRCZ). Informed written consent was obtained from the respondents.

### Sampling

Formative research began in July 2012. Mothers with infants less than one year were interviewed using qualitative techniques. The two districts had similar health indicators such as prevalence of stunting. We also found that there were several information and education materials (IEC) developed by the Ministry of Health and Child Care for mothers to use but were frequently not being used. We found a gap where mothers have many questions throughout the lifetime of their babies that require solutions. Messages with most asked questions and answers were developed. These were translated to local language (Shona and Ndebele) and channels of delivery to reach mothers were identified. We used geographical and administrative boundaries to identify clusters and 79 clinics and polyclinics were eligible for randomization. Consultation with local authorities indicated that the district was subdivided into constituencies and clinics in each of the constituencies were grouped and used as clusters. Therefore a cluster was a group of neighbouring clinics in a constituency. We selected clinics at the centre of clusters leaving a buffer zone around them. The clinics and polyclinics were then randomized separately into eight clusters. The four clusters received training in cIYCF intervention while the other four clusters were controls. Using a two by two factorial design, each of the two clusters were randomized a second time to receive a newsletter while the other two acted as controls (Table 1).

**Table 1:** **Two by two factorial design: effect of mother-based promotion of exclusive breastfeeding on duration and severity of diarrhoea and pneumonia**

	**cIYCF Training**	
**Newsletter**	**Trained**	**Not trained**
Received newsletter	Trained + received newsletter: 91 mother-infant pairs	Not trained + received newsletter: 92 mother-infant pairs
No newsletter	Trained + no newsletter: 95 mother-infant pairs	Not trained + no newsletter: 93 mother-infant pairs

To allocate one of the two clusters to either of four groups, random numbers were generated from Stata 10 by an independent statistician. All infants delivered during the recruitment period in each cluster were eligible for randomization. Participants were randomized within their clusters. Informed and written consent was sought from mothers of infants during recruitment. Because of the nature of our study, recruitment was conducted at cluster level by nurses and VHWs who were not aware of the hypothesis being tested. However, study participants were not blinded to their allocation but every effort was made to ensure that another research assistant assessing diarrhoea duration, and incidence of pneumonia was unaware of the group assignments, was unaware of the hypothesis being tested and analysis of primary and secondary outcomes was only done after 20 weeks (Figure 1).

### Intervention activities

At the control clusters, routine services were provided according to the Zimbabwe Ministry of Health and Child Care (MOH&CC) national policy. According to the policy, health care workers are required to counsel mothers on EBF for the first six months of life at every given opportunity. They are supposed to attend to mothers with breastfeeding difficulties and arrange for follow up if necessary. Where there are breastfeeding support groups mothers should be referred to these groups in the communities where they stay. Also mothers continue to be counselled and supported on prevention of mother to child transmission of HIV. Health care workers in control areas were not informed about their participation as comparison group.

At the intervention clusters, mothers received the routine services provided according to the MOH&CC national policy as outlined in the control group. In addition, from August 2012 to November 2012, 214 village health workers (VHWs) were trained on cIYCF using training materials adapted from the community infant and young child feeding counselling package developed by UNICEF. The training was conducted in clusters and covered a period of five days per group. From December 2012 to February 2013, mothers who delivered in selected clusters received educational material (a breastfeeding newsletter) in one group while the other group received no newsletter. The newsletter was given to mothers at enrolment by the nurses at health facilities and VHWs within the community.

The title of the newsletter was “Exclusive breastfeeding for the first six months of the baby’s life: the perfect food.” The contents included dates for the immunizations according to the new child health card, messages on feeding only breast milk for the first six months, frequency and duration of breastfeeding as well as most asked questions and answers. Short and concise key messages on immediate breastfeeding after birth, feeding only breast milk for the first six months of life, and breastfeeding the infant day and night, at least eight to ten times in 24 hours were imparted. Specific foods and fluids given to non-exclusively breastfed infants, such as water, gripe water, cooking oil and *muti* (herbal mixtures) were targeted in the newsletter to explain their lack of benefit and possible negative effects on child’s health.

The newsletter also advertised EBF, where the statements; “Who is going to be our best mother at 14 weeks?” and also “remember, to win just give your baby breast milk only for the first 6 months” were used. There was also a section for feedback information. The section on advertising breast milk was meant to idealize EBF so as to encourage mothers to have a sense of perceived competition to be the best mother at 14 weeks. Since we also wanted to test if non-financial incentives influence exclusive breastfeeding among women, a sub group of 30–35 mothers randomly received a newsletter with an extra section of a question that needed to be answered at 14 weeks and a reinforcing statement reminding them of the need to read and understand the contents of the newsletter to stand a better chance to win. During follow up, the interviewer would request to see the newsletter and classify those mother-infant pairs that received a newsletter with an extra section using code 1 and those without code 0. Those with code 1 were included in the sub-group analysis. However, all mothers in the intervention sites received a t-shirt with breastfeeding messages at the end of assessment (at 20 weeks). The newsletter sought to empower the mother to take full control of her child’s health issues. This intervention was mother-based because it articulated the mother as the agent of change and this was achieved through the newsletter to the mother.

Follow up of mother-infant pairs was done after 14 and 20 weeks of delivery and took place between April 2013 and July 2013. At 14 weeks the trained nutritionist and nutrition assistants followed up mothers at selected clusters on scheduled visits. Mothers who did not turn up to the health facility on scheduled visits were followed to their homes to ascertain EBF rates and occurrence of illness using standardized questionnaires. The mother was asked if she had given the baby any liquids or solids in addition to breast milk in the past 24 hours (24 hour dietary recall) and or seven days, but 24 hour recall was used in analysis. Mothers were also asked if the child had recurrent episodes of diarrhoea and pneumonia in the past seven days, if the child had suffered from diarrhoea in the past seven days and for how long. The medication given for treatment of diarrhoea was also recorded. Child health cards were checked to see if the child had received pentavalent 3 at 14 weeks.

At 20 weeks mothers were visited at home and the same questions asked at 14 weeks were used. Mothers were given a follow up newsletter at six months with information on how to sustain breastfeeding while giving timely and appropriate complementary foods up to two years or beyond.

### Operational definitions

- Exclusively breastfed infant was defined as an infant who received only breast milk for the first six months of life and not even water except for medicines prescribed by a doctor or nurse [18,19]. In this study any deviation before the age of 14 weeks and 20 weeks was not classified as EBF.
- Any infant who received breast milk as the main source of nourishment but also received other liquids in addition before the age of six months was predominantly breastfed and any infant who received both breast milk [19] and other foods before the age of six months was classified as mixed fed.
- Diarrhoea was the passage of three or more frequent loose or liquid stools per day or more frequently than is normal for the child [19,20].
- Severe diarrhoea was any infant who presented with signs of dehydration (dry sunken eyes, very dry mouth, extreme thirst and little or no urination) and if the child reported to the health facility any child who received intravenous fluids.

### Outcome variables

The primary outcomes were duration and severity of diarrhoea as well as incidence of pneumonia and diarrhoea at 14 and 20 weeks. Diarrhoea and pneumonia incidence were measured as recurrent or new episodes of the illness within 2 to 3 days [21,22]. Primary outcomes were chosen because they are measurable and their ability to measure the effectiveness of an intervention on duration of diarrhoea.

Our secondary outcomes were exclusive breastfeeding prevalence and diarrhoea prevalence at 14 and 20 weeks as well as vaccination uptake (ascertained using age appropriate receiving of pentavalent 3 at 14 weeks). The prevalence of breastfeeding was assessed using 24 hour recall while for prevalence of diarrhoea, seven day recall was used.

### Data analysis

The sample size was calculated on the basis of methods appropriate for cluster randomized controlled trials. A sample size of 85 participants per intervention group was expected with 80% power, a two-sided 5% significance level, assuming an attrition rate of 10% and an estimate of k = 0.25 borrowed from a study in India to detect a 19% difference in the percentage increase of children who are exclusively breastfed up to six months of age after exposure to intervention [19].

Epi Info version 7 was used for data capturing while SPSS version 16 and Stata 11 were used to analyse data. Since randomization was by clinic, we adjusted for cluster randomization. Two-way factorial ANOVA was used to assess the relationship between two independent variables and the dependent variable. For the exclusive breastfeeding, diarrhoea, pneumonia outcomes; we calculated proportions, adjusted odds ratios, relative risks and their 95% confidence interval. A p - value of less than 0.05 (<0.05) was judged as significant. Sub-group analysis was conducted and reported separately. All analysis was by intention-to-treat.

## Results

### Participants

**Figure 2:**
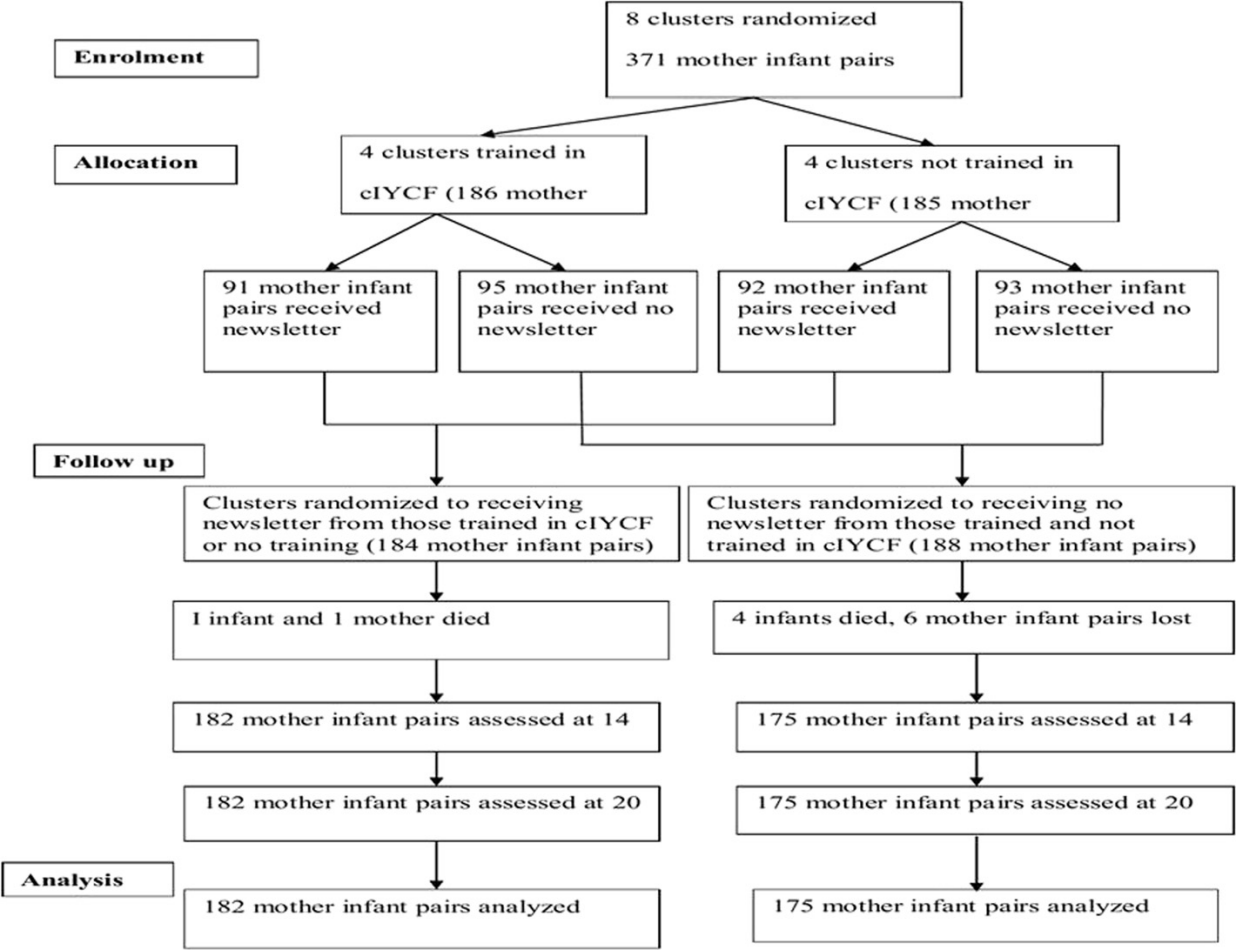
**Participants flow chart.**

The participants flow chart (Figure 2) shows the trial profile of the randomized trial. All the eight clusters received their allocated intervention. The 371 infants born between December 2012 and February 2013 were identified; 186 from the intervention communities that were trained in cIYCF and 185 from the control communities that were not trained in cIYCF. All the groups were presumed to be having similar characteristics and the dropout rate was 3.6%. The major reasons for loss to follow up were neonatal death, husbands declining consent given by their wives and change of place of residence in urban settings. There was no significant difference in the distribution between males and females. There was also no association between independent variables and gender of children. 357 mother infant pairs (excluding twins) were available for analysis in all the clusters.

### Demographic characteristics of mother-infant pairs

The common occupations for men both in the rural and urban settings was informal employment mostly gold panning while majority of women were unemployed. The majority (>90%) of infants were delivered at institutions and breastfeeding was initiated within an hour of delivery. The mean age of mothers ranged from 26.6 to 27.0 years and the majority of mothers had attained secondary education (range from 60.1% to 70.9%) (Table 2).

**Table 2:** **Socio-demographic characteristics of mother-infant pairs, Midlands Province, Zimbabwe, 2013**

**Variable**	**Characteristic**	**cIYCF**	**No cIYCF**	**Newsletter**	**No Newsletter**
		**(n = 178)**	**(n = 179)**	**(n = 182)**	**(n = 175)**
Sex	Female	89 (50.0)	9 8 (54.7)	88 (48.3)	99 (56.6)
	Male	89 (50.0)	81 (45.3)	94 (51.6)	76 (43.4)
Birth place	Facility	163 (91.6)	163 (91.1)	165 (90.6)	161 (92.0)
	Home	15 (8.4)	16 (9.9)	17 (9.4)	14 (8.0)
Breastfeeding initiation	<1 hour	64 (67.4)	70 (75.3)	48 (57.8)	50 (60.2)
	>1 hour	31 (32.6)	23 (24.7)	35 (42.2)	33 (39.2)
Mother’s age	Mean yrs (SD)	26.6 (6.4)	27.0 (6.5)	26.9 (5.9)	26.8 (6.9)
Education	Primary	23 (12.9)	21 (11.7)	15 (8.2)	29 (16.6)
	Secondary	155 (87.1)	158 (88.3)	167 (91.8)	146 (83.4)
Marital status	Married	162 (91.0)	160 (89.4)	174 (95.6)	148 (84.6)
	Other	16 (6.2)	19 (2.1)	8 (4.4)	27 (15.4)
Residence	Rural	97 (54.5)	98(54.7)	81 (44.5)	81 (46.3)
	Urban	81 (45.5)	81 (45.3)	101 (55.5)	94 (53.7)
Employment	Formal/informal	46 (25.9)	44 (24.6)	34 (18.7)	56 (31.9)
	Unemployed	132 (74.1)	135 (75.4)	148 (81.3)	119 (68)
HIV status	Negative	146 (81.9)	146 (81.6)	149 (85.5)	143 (81.7)
	Positive	32 (18.1)	33 (18.4)	33 (18.1)	32 (18.3)

### Effect of intervention on exclusive breastfeeding

At 14 weeks, 356 (99.7%) and at 20 weeks 355 (98.3%) of infants were still breastfeeding. Both the newsletter (p < 0.0001) and cIYCF training (p < 0.0001) had positive effects on exclusive breastfeeding. More infants were exclusively breastfed in the groups that received intervention. The exclusive breastfeeding rates were higher, 147 (81.6%) in the group that received a newsletter compared to 67 (38.3%) in the group that received routine services only at 14 weeks (p < 0.001). On the other hand, 118 (66.7%) of infants with mothers residing in communities that received cIYCF training only were exclusively breastfed. Furthermore, infants of mothers who received the newsletter (RR = 2.12, 95% CI; 1.73, 2.58) and infants of mothers who resided in communities trained in cIYCF (RR = 1.31, 95% CI; 1.10, 1.55) were more likely to be exclusively breastfed compared to their counterparts at 14 weeks. However, at 20 weeks infants of mothers who received a newsletter were 3.1 (95% CI; 2.31, 4.07) times more likely to be exclusively breastfed compared to their counterparts (Table 3).

**Table 3:** **Effect of intervention on exclusive breastfeeding, Midlands Province, Zimbabwe**

	**Exclusive breastfeeding**		**Total (n = 357)**	**Relative Risk**	**95% CI**
	**Yes**	**No**			
**At 14 weeks**					
cIYCF only	118 (66.7)	59 (33.3)	177	1.30	1.09, 1.56
No cIYCF	91 (50.8)	87 (49.2)	179	0.66	0.51, 0.86
Newsletter only	146 (81.0)	35 (19.0)	181	2.23	1.81, 2.75
No newsletter	63 (36.0)	111 (64.0)	175	0.30	0.22, 0.42
**At 20 weeks**					
cIYCF	90 (50.8)	87 (49.2)	177	1.20	0.96, 1.50
No cIYCF	76 (42.4)	103 (57.6)	179	0.85	0.70, 1.04
Newsletter	129 (71.3)	52 (28.7)	181	3.37	2.49, 4.55
No newsletter	37 (21.1)	130 (78.9)	175	0.37	0.29, 0.47

In the linear regression model, both the cIYCF training and newsletter were independent predictors of exclusive breastfeeding at 14 weeks (p < 0.0001) while at 20 weeks only the newsletter remained a significant predictor of EBF and worked better in increasing EBF compared to cIYCF alone (p < 0.0001). Also 67% of infants who were not exclusively breastfed might have been exclusively breastfed if their mothers had received the newsletter.

### Effect of intervention on duration of diarrhoea

A two- way ANOVA was conducted to examine the effect of cIYCF and the newsletter to the mother on duration of diarrhoea at 14 and 20 weeks. At 14 weeks, there was a statistically significant interaction between cIYCF training and the newsletter (p = 0.026). The mean duration of diarrhoea for infants with mothers who resided in communities that received both interventions was 2.9 ± 0.94 days while those that resided in communities where mothers received a newsletter alone was 3.6 ± 1.19 days compared to 5.2 ± 1.15 days, in infants of mothers who received no intervention. Thus the effect of cIYCF training is modified by whether mothers of infants received a newsletter. At 20 weeks, there was no statistically significant interaction (p = 0.783) but the newsletter worked better than the cIYCF training in reducing mean duration of diarrhoea (p = 0.020) (Table 4). In a one way ANOVA, there were no statistically significant differences in mean duration of diarrhoea between infants of mothers that received a newsletter only and infants of mothers who received both interventions (p = 0.978).

**Table 4:** **Mean duration (Days) of diarrhea among infants of mothers who received cIYCF plus newsletter, cIYCF training only, newsletter only and or routine services only, Midlands Province, Zimbabwe, 2013**

**Variable**		**Diarrhoea n (%) (n = 357)**		**Mean (SD) days**	**p-value**
**Mean duration (days) of diarrhea at 14 weeks**					
cIYCF trained	Yes	11 (6.2)	178	2.9 (0.94)	<0.0001
	No	10 (5.6)	179	3.1 (0.88)	
Received newsletter	Yes	13 (7.1)	182	3.6 (1.19)	0.005
	No	20 (11.4)	175	5.2 (1.15)	
cIYCF trained and received newsletter					0.026
**Mean duration (days) of diarrhea at 20 weeks**					
cIYCF trained	Yes	5 (2.8)	178	4.8 (0.8)	0.361
	No	36 (20.1)	179	5.7 (1.4)	
Received newsletter	Yes	14 (7.7)	182	5.1 (1.2)	0.022
	No	38 (21.7)	175	6.0 (1.8)	
cIYCF trained and received newsletter					0.764

### Effect of intervention on severity of diarrhoea

The information obtained during follow up at 14 and 20 weeks showed no effect for the newsletter (p = 0.182) and cIYCF training (0.315) on severity of diarrhoea. However, the infants of mothers who received a newsletter were less likely to have diarrhoea at 20 weeks compared to infants of mothers who did not (RR = 0.24, 95% CI; 0.15, 0.38). On the other hand, the infants of mothers who resided in communities trained in cIYCF were also less likely to have diarrhoea but the association was not statistically significant (RR = 0.78, 95% CI; 0.48, 1. 10). Compared to infants of mothers who received routine services only, infants of mothers who received a newsletter were less likely to have recurrent episodes of diarrhoea at 20 weeks (RR = 0.24, 95% CI; 0.15-0.38) (Table 5). Infants of mothers in the intervention communities were more likely to seek treatment for diarrhoea compared to no intervention communities, however the association was not statistically significant. All infants with diarrhoea in both intervention and non-intervention sites who reported to the facility received ORS and Zinc Sulphate according to the Zimbabwe Integrated Management of Neonatal and Childhood Illnesses guidelines (IMNCI guidelines). The protective efficacy of the newsletter was 76% during the first 20 weeks of life. In summary in the two way ANOVA, the newsletter showed a significant effect on the incidence of diarrhoea at 20 weeks (p = 0.021).

**Table 5:** **Effect of intervention on incidence of diarrhoea in the first 20 weeks of life, Midlands Province, Zimbabwe, 2013**

**Group**	**Diarrhoea**		**N**	**Relative risk**	**95% CI**	**Preventive fraction (%)**
	**Yes**	**No**	**357**			
Trained	41	137	178	0.78	0.55, 1.10	
Not trained	52	127	179	1.09	0.96, 1.24	
Newsletter	19	163	182	0.24	0.15, 0.38	76%
No newsletter	75	100	175	1.57	1.37, 1.81	

### Effect of intervention on duration of pneumonia

There was no interaction between the newsletter and cIYCF training on the duration of pneumonia (p = 0.975). At 14 weeks, the newsletter was more effective on duration of pneumonia (p = 0.010) and, receiving a newsletter was more effective irrespective of whether the mother resided in communities trained in cIYCF or not. Furthermore, the mean duration of pneumonia among infants of mothers who received newsletter was 3.8 ± 1.79) days, compared to 6.7 ± 3.65) days among infants of mothers who did not receive the newsletter. In addition, Table 6 shows that there was a statistically significant difference between groups at 20 weeks as determined by one way ANOVA (p < 0.0001). A pair wise comparison using a Tukey post-hoc test revealed that the mean duration of pneumonia was significantly lower among infants of mothers that received a newsletter only (3.6 ± 1.5 days, p = 0.001) and among infants of mothers of mothers that received both the newsletter and resided in communities trained in cIYCF (3.7 ± 1.7 days, p = 0.001) compared to infants of mothers that resided in communities trained in cIYCF only (6.5 ± 3.0 days, p = 0.948). There were no statistically significant differences between infants of mothers that received a newsletter only and infants of mothers who received both the newsletter plus resided in communities trained in cIYCF (Table 6).

**Table 6:** **Mean Duration (Days) of pneumonia among infants of mothers who received cIYCF and newsletter, cIYCF training, newsletter only, and or routine services only, Midlands Province, Zimbabwe 2013**

**Group**	**N**	**Mean (SD) days**	**p- value of pairwise contrasts with;**		
	**357**		**Routine services only**	**cIYCF only**	**Newsletter only**
**Mean duration of pneumonia at 20 weeks**			*X*_2_ P value < 0.0001		
cIYCF only		6.5 (3.0)	0.948		
Newsletter only		3.6 (1.5)	0.001	0.003	
Newsletter + cIYCF		3.7 (1.7)	0.001	0.006	0.999

In addition, infants of mothers who received a newsletter (RR = 0.26, 95% CI; 0.15-0.46) and infants of mothers who resided in communities trained in cIYCF (RR = 0. 48, 95% CI; 0.30-0.78) were less likely to suffer from pneumonia compared to infants of mothers who did not at 20 weeks. The preventive efficacy of the newsletter at 20 weeks was 74%.

### Effect of intervention on uptake of immunization (Pentavalent 3)

The main effect of the newsletter on uptake of pentavalent 3 at 14 weeks was statistically significant (p = 0.037). The proportion of infants of mothers who received a newsletter, 141 (77.5%) received age appropriate immunization compared to 113 (62.1%) among infants of mothers who did not receive a newsletter. Thus, infants of mothers who received the newsletter (RR = 1.21, 95% CI; 1.05, 1.38) were more likely to receive age specific immunization than infant of mothers who received no newsletter (RR = 0.88, 95% CI; 0.77-1.00).

## Discussion

Our findings indicate that a combined community infant and young child feeding training and a newsletter reduced the mean duration of diarrhoea by a remarkable 2.3 days at 14 weeks compared to control areas. The findings also showed that 76% of infants who had diarrhoea might have been prevented if their mothers had received both interventions at 14 weeks. No other study has measured the effect of mother- based promotion of exclusive breastfeeding intervention in Zimbabwe using a factorial design.

On the other hand, our study demonstrates that providing timely and accurate information to mothers is effective, fundamental and promotes exclusive breastfeeding. Additionally, mother-based promotion of exclusive breastfeeding results in an increase in exclusive breastfeeding rates, reduced duration of diarrhoea and pneumonia as well as significant increase in age specific immunization through the use of a newsletter.

The findings of this study are however unique in that the cue to action was achieved through an approach that is potentially replicable, sustainable and was delivered through existing routine health services in primary health care settings. The result that the newsletter works better than community infant and young child feeding training is not in accord with any previous studies as we did not find a similar approach in literature. We are also not aware of other published studies that have evaluated the effects of mother-based promotion of exclusive breastfeeding, but the approach may be similar to other studies where letters were used to increase adherence to tetanus booster vaccination among adults [23] and letters that were used in improving immunization coverage [24]. In Tanzania, a Cochrane systematic review showed that the use of letters was effective in improving male participation in the PMTCT programme [25]. Nevertheless, the design or even the context was not similar to our study.

Thus, relative to no intervention, the use of both the newsletter and cIYCF training for village health workers resulted in a more than double absolute mean decrease in the duration of diarrhoea while the newsletter remained effective even up to 20 weeks. The possible explanations for this finding is that; village health workers trained in cIYCF track mothers before and soon after delivery to record them in their registers. So during this period they are more likely to counsel mothers for exclusive breastfeeding. On the other hand, firstly, the newsletter is a marketing tool that ensures that women receive complete, accurate, timely, and consistent information on breastfeeding which is fundamental for any program promoting exclusive breastfeeding [18-20]. It demonstrates the feasibility of disseminating breastfeeding messages directly to the intended users in the developing world. Thus if the media and various communications target optimal breastfeeding, it is possible to achieve universal exclusive breastfeeding in Zimbabwe.

Secondly, a newsletter has the properties that can help women to sort the myths surrounding breastfeeding. It also helps women to understand the benefits of breastfeeding without misinformation or mixed messages while ensuring that women receive accurate, complete and consistent messages to protect, promote and support breastfeeding [26].

Thirdly, the newsletter acted as a reference material that was used by mothers at home to solve common breastfeeding problems that were highlighted in some studies as a barrier to exclusive breastfeeding [27-29]. Furthermore, since the newsletter contained information on most frequently asked questions and answers, how to overcome breastfeeding difficulties aided in improving breastfeeding practices thereby impacting on diarrhoea and pneumonia duration [30].

Our findings are consistent with other studies that evaluated the effects of community-based interventions on EBF [19,31-34]. Unanimous evidence shows that interventions that increase exclusive breastfeeding have positive effects on reducing duration of diarrhoea and pneumonia [35-39]. The reduction then impacts on severity of diarrhoea and the ultimate goal to reduce child/infant mortality can be achieved [17,20]. Another study conducted in Dhaka, Bangladesh showed that deaths from diarrhoea and pneumonia could be reduced by a third if infants were exclusively breastfed for first six months [21,33,39-41].

Nevertheless, the findings of this study also probably indicate the less effectiveness of the cIYCF training beyond 14 weeks after delivery. The possible implication of our results is that with cIYCF alone, universal exclusive breastfeeding or reaching the target of 90% EBF up to six months will not be possible in Midlands Province.

There are several explanations to our findings; the effectiveness of cIYCF training depends on the full coverage of the trainings in all facilities and their communities and thus uses a dose response relationship. In our study setting, some clusters were trained but full coverage was not achieved. This might have impacted heavily on the village health worker: mother ratio which was estimated at 1 VHW: 365 mothers, with most mothers not receiving the benefits of the trainings.

A report in *The Lancet* of a randomized trial comparing the effects of hospital-based system and community-based system intervention providing ten postnatal home visits found that home visits significantly increased the chances of exclusive breastfeeding [32,33,42]. Thus in our setting, it might be possible that home visits were only being conducted soon after delivery and probably up to three months while beyond 14 weeks the frequency became insignificant. This is of great concern and thus calls for other interventions that help to sustain EBF up to six months. Thus a breastfeeding newsletter bridges the gap as it is given directly to the mother. Providing consistent information is an enabling environment for sustainable EBF up to six months.

Another study have shown that the use of trained peer counsellors on the pay roll was more effective compared to volunteers [32,34]. The use of volunteers is the most likely scenario in our case and hence it is less effective. This might be because the effectiveness of cIYCF depends on motivated peer counsellors or volunteers, since the approach relies on training peer counsellors or volunteers to impart knowledge to mothers. Several studies elsewhere suggested the implementation of cIYCF at scale, intensive follow up and supportive supervision, as well as routine monthly meetings or non- financial incentives to peer counsellors as improving the effectiveness of cIYCF trainings.

In this study, the lack of association with severity of diarrhoea can be explained by the fact that, though not exclusive, over 98% of infants in all groups were being breastfed. Given that continued breastfeeding reduces dehydration, few infants developed severe diarrhoea and thus probably the sample size was not adequate to determine meaningful differences. It is also possible that treatment efficacy of Zinc Sulphate and oral rehydration solution played an important role in treatment and prevention of dehydration. Similar findings were reported in an educational intervention where village and community health workers were trained in community infant and young child feeding showed an increase in exclusive breastfeeding but no association with severe diarrhoea [22,35,36,39,43].

Cluster randomized trials are also prone to bias. We noted some differences in employment status and knowledge on exclusive breastfeeding among infants of mothers. In addition, in the cluster that received both interventions, an organization called Zvitambo was also following up breastfeeding mothers. However, we do not think that the highlighted factors can contribute to the differences noted in diarrhoea and pneumonia duration, but deserve further investigation.

It is also possible that information highlighted in the newsletter that was given to a sub-group of mothers in intervention communities contaminated intervention sites and reinforced the motive to read and understand the contents so that one stands a chance to win. In so doing, mothers understood the benefits and dangers of not breastfeeding exclusively. In tandem with the Health Belief Model, a theory widely used in preventive health behaviour; because of the perceived benefits, mothers were more likely to take action. Perceived benefits are a determinant of preventive health actions [44]. However, the role of chance could not be ruled out as a possible explanation for this finding.

The use of a breastfeeding newsletter and a newsletter plus cIYCF can be generalized to other types of preventive care, since this approach was borrowed from the promotion of healthy lifestyles in Japan through the Healthy Obihiro 21. However it would be an important area for future research. Since the intervention depended on ability to read, findings cannot be generalized to illiterate communities.

Some limitations of our study included observation bias in reporting especially mothers of infants in the intervention communities tended to over report exclusive breastfeeding and under report illnesses. This was overcome by the use of trained nutritionist and nutrition assistants.

## Conclusions

There is sufficient evidence to conclude that the effect of a breastfeeding newsletter vs. no newsletter on duration of diarrhoea at 14 weeks is probably different for those residing in communities trained in cIYCF or not. At 20 weeks the newsletter worked better for both duration of diarrhoea and pneumonia compared to cIYCF training alone. In addition, both the newsletter and cIYCF training had positive effects on exclusive breastfeeding at 14 weeks, but the newsletter worked better at 20 weeks compared to cIYCF. Therefore both interventions can be implemented and up scaled in combination to realize the maximum benefits in promoting, protecting and supporting breastfeeding. We recommend a combined community and mother-based intervention using a breastfeeding newsletter to be up scaled with adequate follow up of the trained cadres.
